# The fire hazard preparedness of special needs schools in the North West Province, South Africa

**DOI:** 10.4102/jamba.v16i1.1669

**Published:** 2024-09-30

**Authors:** Tlou D. Raphela, Ndivhuwo Ndaba

**Affiliations:** 1 Department of Disaster Management Training and Education Centre for Africa, Faculty of Natural and Agricultural Sciences, University of the Free State, Bloemfontein, South Africa

**Keywords:** preparedness, mitigation, fire hazard, injury mechanisms, people with disabilities

## Abstract

Fire disaster preparedness has been reported as critical in schools, especially for schools that have learners with special needs. This study, therefore, through a quantitative research approach, collected data from 88 participants across nine special needs schools (SNS) in the North West Province of South Africa to assess the fire preparedness of these schools. Data were collected using a structured questionnaire. The study applied descriptive and inferential statistics using R Statistical Software package to analyse questions that gauged the fire preparedness of the sampled schools. Four generalised linear and one ordinal regression models were applied to the data and found statistically significant differences for some predictors across the four applied GLMs. Significant results were found for the school fire safety management model applied to the Disaster Preparedness Strategy framework (χ^2^ = 206.7; *p* = 0.01; *R*^2^ = 90.5%). However, a significant difference was only found in the question of early warning systems across the eight predictor variables for the framework.

**Contribution:**

The maintenance and recordkeeping model showed significant results in terms of record maintenance and the frequency of fire safety equipment inspections. While compliance with specific fire safety regulations is essential, broader engagement and continuous improvement in early warning systems are necessary for a more resilient disaster preparedness strategy. This study addresses a critical gap in understanding and improving fire hazard preparedness in these schools. The authors therefore recommend intervention from the authorities to assist these schools to prepare for fires.

## Introduction

Fire-related burn injuries are a disproportionately prevalent and critical health risk in Africa, with a significant impact on children, the elderly and individuals with disabilities (eds. Sherry et al. 2024). Approximately 1 000 African children under the age of 5 years succumb to fire-related injuries each year (Torpy, Lynm & Glass 2009). According to Davies, Du Toit and Hlela (2024), the rate of child fatalities resulting from burns is currently more than seven times greater in low-income countries in Africa compared to high-income countries, representing one of the most substantial disparities in injury mechanisms internationally. The number of lives lost, and injuries caused by fires in African countries is deeply concerning. The Fire Protection Association of Southern Africa (FPASA) reported in 2022 that 645 individuals lost their lives because of structural fires in Southern Africa in 2021 (Essack & Ilemobade 2023). This underlines the urgency of implementing effective fire prevention and safety measures in the region to protect lives and property.

South Africa faces one of the highest rates of injury-related deaths and disabilities because of fires (Peck 2011). Children with special needs are particularly vulnerable during fire emergencies across the world (Ronoh 2017). Therefore, the assessment and awareness of fire safety education by teachers and learners in special needs schools is recommended (Kong, Shin & Seo 2017). Murray (2011) concurs that the issues for children with special needs in fire disaster preparedness are neglected and the literature on these studies is scanty. Boon et al. (2011) also conducted a study with Australian schools and found that the needs of learners with special needs have not been adequately reflected in school fire emergency plans from a postal survey across Australia. In Africa, Raphela and Ndaba (2024) reported that the safety and well-being of learners with special educational needs in South Africa remain a paramount concern. In recognition of the increased fire risks in these schools, the US National Fire Protection Association (Ronoh et al. 2007) has created a personal emergency evacuation planning tool for school students with special needs (Ronoh 2017).

It is important to recognise that while deaths are a significant concern, they represent just one aspect of the problem as far as fire disasters are concerned. As highlighted by Torpy et al. (2009), for every individual who loses their life because of fire burns, many more are left with enduring disabilities and permanent scars. Moreover, the consequences of surviving fire-related injuries can be profound. Individuals who survive such incidents often experience prolonged hospitalisation, enduring disfigurement and disabilities (Patterson et al. 1993). These outcomes can lead to social stigma and rejection, further compounding the challenges faced by fire survivors (Christianson & Christianson 2020). Statistics South Africa credits 2246 deaths to fire smoke and flames in their research of the distribution of mortality owing to other external causes of unintentional injury in 2021 (Christianson & Christianson 2020; Essack & Ilemobade 2023).

South Africa has shown its commitment to providing quality, equitable and inclusive education through various legislations (Mantshiu 2017). However, despite these legal provisions, children with disabilities continue to face marginalisation, primarily because of the absence of proper fire safety measures that enhance preparedness in SNS across many provinces in South Africa (Makwela & Smit 2022). This lack of fire safety measures in schools for learners with special needs poses a significant challenge, demanding immediate intervention to ensure that learners are not exposed to hazardous and life-threatening conditions (Geldenhuys & Johnson 2019). The inadequate fire safety and preparedness measures in SNS in South Africa not only present a physical threat to the learners but also send a signal that they are not recognised or valued by South African communities. As a result of the legacy of apartheid, often poor black South African learners are disproportionately denied access to a safe learning environment (Badat & Sayed 2014). As noted by the South African Human Rights Commission, poor black learners with special needs experience a compounded form of marginalisation, highlighting the urgent need to address this issue and ensure the safety and well-being of all learners, regardless of their background or abilities (Geldenhuys & Johnson 2019). These challenges cannot be used as a justification for failing to plan for and mitigate the imminent risks that have already resulted in tragic consequences for learners, particularly in the North West Province. For example, in 2015, at the North West School for the Deaf, a devastating fire led to the fatal injuries of three female learners aged 16, 17 and 18 years (Tshatshu 2016).

Additionally, 23 female learners were injured while attempting to escape the fire by jumping from the first to the ground floor of a building (Geldenhuys & Johnson 2019). These deaths indicate the serious risks posed by inadequate or improper safety measures and preparedness in SNS throughout the North West Province. These incidents are not isolated; they are part of a concerning pattern of fatal incidents involving vulnerable learners. These include the Christiana School for the Blind, where three blind students lost their lives in a fire in 2010 (Christianson & Christianson 2020). The other traditional school in the North West Province, Hoër Volkskool in Potchefstroom, also experienced a fire outbreak in the male learners’ hostels in 2015 (Montsho 2015). The frequency of such occurrences in schools in general is alarming and necessitates urgent attention and action to ensure the safety and well-being of all learners in South Africa. This study sought to answer the question of how prepared SNS in the North West Province of South Africa are for fire hazards.

Even though fires are a major hazard, a significant majority of schools worldwide lack adequate fire safety measures (Stokes & Johnson 2017; Torpy et al. 2009). This lack is even more pronounced in SNS, where fire safety management remains particularly overlooked (Gichuru 2013). Additionally, there is a scarcity of scientific literature addressing fire safety management specifically tailored to SNS (Raphela et al. 2024). Therefore, this study assessed the preparedness of SNS in the North West Province of South Africa for fire hazards by running four models and by applying the Red Cross Red Crescent Preparedness Strategy framework to the study. This framework includes nine elements of preparedness that were turned into eight independent variables and one turned into an outcome variable (see the output of the regression model for the questions in Table 1).

**TABLE 1:** Output of regression analysis for the Likert scale questions to measure school fire safety management.

Indicators: Does your school …	Estimates	SE	Wald	*P*
Do hazard, risk and vulnerability assessments?	0.096	1.915	0.003	0.960
Have response mechanisms and strategies for fires?	−0.176	2.537	0.005	0.945
Have a fire preparedness plan?	−0.124	6.311	0.000	0.984
Have fire coordinators?	−0.044	3.469	0.000	0.990
Have an effective information management system for hazards?	0.123	2.129	0.003	0.954
Have early warning systems?	**12.575**	**4.631**	**0.373**	**0.007**
Mobilise resources?	0.222	7.244	0.001	0.976
Engage in public education, training and rehearsals?	0.211	3.498	0.004	0.952

SE, standard error.

Note: Significant parameters are shown in bold.

## Research methods and design

The study design is purely quantitative with a post-positivist philosophical worldview approach and was purposive in nature. The study collected data using a questionnaire with close-ended questions and Likert scale questions developed from the Preparedness Strategy framework of the Red Cross Red Crescent. All nine schools sampled for this study were purposefully selected to suit the criteria for selection for this study (see research design for the criteria). A structured questionnaire was used to collect data from 88 participants from these nine schools. The study administered 122 questionnaires and received 100 responses. Of these, 11 were discarded because some of the critical questions were not answered or the answers did not make sense to the researchers (e.g. some respondents added their responses ‘we are not told anything in this school’ on question 10 and selected it instead of the yes and no closed responses that were predetermined by the study), and one questionnaire was not completed.

The study purposely selected SNS in the North West Province of the country by looking at special needs holistically. The list of the schools targeted for the study included SNS that were generic across the Basic Education system of South Africa, including primary, secondary, middle and high schools sourced from the Department of Basic Education (DBE) database. The Department of Basic Education Central Schools database has a record of 32 SNS in the North West Province.

The study did not select schools that cater to the specific types of special needs, such as schools for the blind, schools for the deaf, or comprehensive special needs schools, as these schools will not use generic preparedness frameworks or strategies. Even though preparedness cuts across any special needs of learners, these schools will have different and specialised preparedness strategies; for example, schools for the deaf will not use fire alarm systems for fire warnings. Furthermore, schools with no historical data and previous research on preparedness interventions were selected, based on the National Disaster Management Centre (NDMC) database. The information about the schools’ criteria was obtained from the DBE and NDMC databases of special needs schools in South Africa.

As the study sampled SNS holistically, only educators and school governing body members were selected. The study targeted two populations: (1) the school; and (2) the respondents, then used purposive sampling to select nine schools and then applied a formula to determine the sample size of the participants. The participants were recommended by the school principals of the sampled schools based on the fact that they are familiar with fire issues in the school and/or have been involved in fire management at some point. However, to be statistically sound and for reproducibility, the authors calculated the study sample size from a total of 178 target populations from the nine schools, comprising 214 educators and 36 members of the school governing body using the following equation:


$$n=N{\left(Z_{\alpha /2}\right)}^{2}/{\left(X_{\alpha /2}\right)}^{2}+Ne^{2}$$ [Eqn 1]

where:

*n* = the sample size,

*e* = the margin of error,

*N* = the population size (178 for this study),

*Zα*/2 = the *Z*-value corresponding to the desired confidence interval.

The study obtained a sample size of ≈ 121.65 because the study is sampling humans the sample size was rounded off to the nearest 10 to 122 from the calculated ≈121.65 and could only use 88 responses from the questionnaires for this study (see section on Results). This study acknowledges the limitations of interviewing educators and members of the school governing bodies only and excluding the learners. However, this was done to comply with the ethics requirement of the country for data collection from minors and learners with special needs. Special needs schools also have learners with mental health-related issues, such as autism and Down syndrome, who are regarded as the most vulnerable (Turner 2020). Interviewing such learners is subjected to a series of ethical permissions from the university that can be obtained; but because this study was for a Master’s degree project, obtaining this series of ethical clearances was going to delay the project that was self-funded and has a residency period at the university. Furthermore, the reliability of the information from those learners (potential participant learners with autism and Down syndrome) is in most cases questionable according to studies (Rasmussen et al. 2001; Siklos & Kerns 2006). Indeed, the study opted to exclude all learners as participants to avoid discrimination in terms of their special needs.

Data validity and reliability were tested by first piloting the questionnaire with nine participants in the sample schools before actual data collection, and Cronbach’s alpha was used to test the scale data on the questionnaire.

### Data collection

This study used a structured questionnaire (see Online Appendix 1) developed by the authors, using literature and preparedness frameworks to address the objective of this study. The questionnaire was divided into two sections that assess questions that address fire hazard preparedness; and lastly, a Preparedness Strategy framework was also used to develop questions that will address the school’s fire safety management (see Online Appendix 1).

Data for this study were collected from June to August 2013 in nine different SNS of the North West Province, which were purposefully selected from the DBE and NDMC databases based on the criteria aforementioned. We collected questionnaire data from 122 respondents; however, only 88 questionnaires were used. Our study design needed data from active educators and members of the school governing bodies, as data were collected from schools during the winter months, which are reported to be fire seasons in South Africa (Strydom & Savage 2016). This was to get data from respondents who are potentially on fire alert because of the season.

### Data analysis

Data for this study were analysed using Microsoft Excel and R Statistical software, and the questions on the questionnaire were analysed descriptively using Microsoft Excel and inferentially using R Software. This study first determined the fire hazard preparedness of the school by asking respondents questions that determined what fire hazard preparedness entails, including training for staff and learners, maintenance and recordkeeping, staff knowledge of protocols and fire compliance using four general linear models (GLMs) (see Questionnaire).

These tests were applied because training, maintenance and recordkeeping, knowledge of protocols and compliance in the context of fire are important variables for fire disaster preparedness (Titko & Ristvei 2020). The study after reviewing the literature and South African standards to comprehend various knowledge areas about fire safety management in schools (Crawford-Brunt 2022; Hassanain et al. 2022; Nyagawa & Anangisye 2023; OHS Act 85 of 1993; SANS 10139); decided to adopt a Disaster Preparedness Strategy framework developed by the Red Cross Red Crescent Society for Training for Fire Safety Management in school facilities. The framework comprises nine elements which were turned into questions for this study as follows: (1) hazard, risk and vulnerability assessments; (2) response mechanisms and strategies; (3) preparedness plans; (4) coordination; (5) information management; (6) early warning systems; (7) resource mobilisation; (8) public education, training and rehearsals; and (9) community-based disaster preparedness.

Disaster preparedness is a broad concept that describes a set of measures that minimise the adverse effects of a hazard including loss of life and property and disruption of livelihoods (Staupe-Delgado 2019). Therefore, to measure school preparedness strategies, the study developed questions using the Disaster Preparedness Strategy framework for the questionnaires, some of which were used as preparedness indicators to determine the level of fire safety in sampled schools using the nine elements of this framework as per the results.

### Statistical analysis

The study assessed fire hazard preparedness for the schools by running four separate generalised linear models (GLMs) as follows: Model 1 – training for staff and learners – included the question ‘whether schools conduct fire evacuation drills for students and staff’ as the outcome variable. The predictor variables included: (1) staff participation in their school’s fire drills; (2) staff confidence in their ability to assist students with special needs during a fire evacuation after receiving the school’s fire preparedness training; (3) staff participation in their school’s fire evacuation training; and (4) the interaction between these factors.

Model 2 – maintenance and recordkeeping – included the question ‘does your school maintain and keep records of fire incidences as they happen?’ as the response variable. The predictor variables included: (1) keeping records of all maintenance; (2) knowledge of the existence of electrical compliance certificates; (3) the frequency of fire safety equipment inspection and maintenance; and (4) the mode of recording participation in fire evacuation drills as predictor variables. Model 3 – staff knowledge of protocol – included questions of: (1) the staff’s knowledge of their responsibility; (2) whether staff will be able to assist with evacuation; (3) the staff’s knowledge of step-by-step fire evacuation procedure specific to their school should a fire break out in their schools; (4) the knowledge of operating a fire extinguisher as predictor variables; and (5) the number of responses to the question of whether staff know protocol should a fire break out in their schools was set as the outcome variable.

Disaster management plans have been reported to be important in preparedness by several studies (Gowing et al. 2017; Skryabina et al. 2017; Tkachuck et al. 2018). Therefore, for model 4, which focuses on fire compliance and planning as the first step to compliance, especially in the context of disaster preparedness, the study then applied the last GLM. This model used the question of whether the participants were aware of the existence of fire safety plans in their schools as the outcome variable and the respondent’s reports on the awareness of the existence or recruitment of fire safety officers or safety committees; the use of any form of flammable chemicals in their school; the storage of flammable chemicals in purpose-made storage; availability of protocols for handling flammable chemicals; registration of these flammable chemicals with the local fire services and the report of whether gas is used at any given point in their school premises as (predictor variables). These questions were asked separately during the survey.

All the GLMs applied for this study were run separately with a glm function and a Poisson distribution, as all the outcome variables for the four GLMs applied were the number of responses.

To measure school fire safety management, the study applied an ordinal regression analysis to the nine Likert scale questions developed from the Disaster Preparedness Strategy framework, which are linked with the indicators used to determine the level of fire safety in schools. Ordinal regression was chosen because the data were not normally distributed even after being log-transformed. The study set the responses to the question ‘Is your school engaged in community-based disaster preparedness?’ as the outcome variable, whereas the responses to the other eight questions were set as the covariates of the model. This was because this study is more focused on the SNS instead of communities.

All data were recorded in Microsoft Excel (Bhanji 2012), and statistical analyses were conducted using R Statistical Software (www.r-project.org, R version 4.4.0, released on 24 April 2024, by the R Foundation for Statistical Computing). Statistical tests were two-tailed, and significance levels were set at *p* ≤ 0.05. All graphs were produced using ggplot2, and the tables were produced using Microsoft Excel. Significance was determined using Wald (χ^2^) statistics for all GLM models. Data for the three models are presented as boxplots and bar charts for model 4 of fire compliance, whereas the data for the preparedness framework are presented as a table.

### Ethical considerations

Ethical clearance to conduct this study was obtained from the University of the Free State, General/Human Research Ethics Committee (GHREC) (No. UFS-HSD2023/0741). In addition, all participants signed the UFS standardised written consent form that had an information sheet about the study before completing the questionnaire.

## Results

To assess the preparedness of the SNS, the study first explored the staff and learners complement (no learners were interviewed for this study; the number was obtained from the principals of the nine schools sampled). The study reported information from the data analysed for 88 questionnaires. The average number of participants per school was 8, with the maximum number of participants being 11. The questionnaires were administered at the nine SNS halls in a focus group type of setting, whereby respondents were gathered in the school halls where they answered the questionnaire individually with the assistance of the research team. The questionnaires administered for the nine SNS were captured and analysed, and the results of this study are based on this analysis.

### Fire hazard preparedness

When assessing fire hazard preparedness, the first model that addressed training for staff and learners showed a statistically significant difference in the question of whether the staff participated in their school’s fire drills (Wald χ^2^ = 27.039; *p* = 0.020); the staff’s confidence in their ability to assist students with special needs during a fire evacuation after receiving the school’s fire preparedness training (Wald χ^2^ = 14.820; *p* = 0.020) and the staff’s participation in their school’s fire evacuation training (Wald χ^2^ = 40.579; *p* = 0.020). However, no statistically significant differences were found for the interactions of the questions: conducting fire drills: participating in fire drills (Wald χ^2^ = 13.534; *p* = 0.256); conducting fire drills: staff’s confidence in their ability to assist students with special needs during a fire evacuation after receiving the school’s fire preparedness training (Wald χ^2^ = 13.534; *p* = 0.196); participating in fire drills: staff’s confidence in their ability to assist students with special needs during a fire evacuation after receiving the school’s fire preparedness training (Wald χ^2^ = 11.754; *p* = 0.6192) and the three-way interaction between conducting fire drills and participating in fire drills: staff confidence (Wald χ^2^ = 11.754; *p* = 0.382).

**FIGURE 1:**
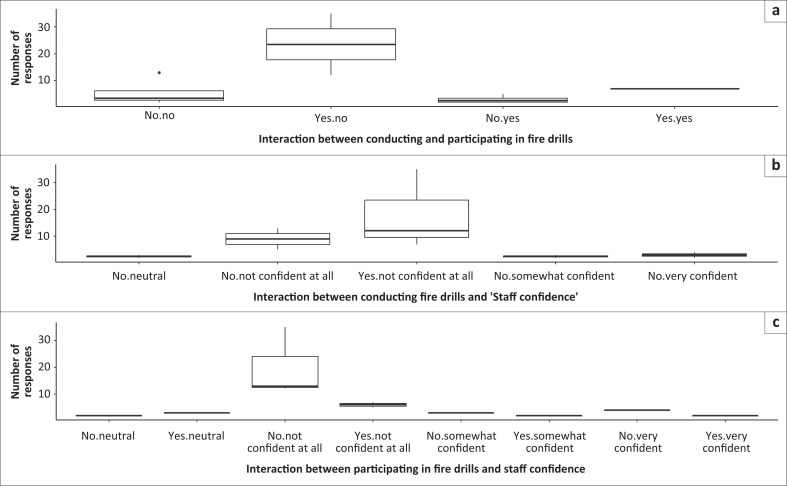
Number of responses for model training for staff and learner: The interaction between conducting and participating in fire drills. (a) the interaction between conducting fire drills and staff confidence; (b) the interaction between participation in fire drills and staff confidence; (c) boxes show the first and third quartiles and medians (solid black line across the box). Whiskers show total range, and dots outside of boxes indicate outliers.

The most frequent responses were ‘yes’ and ‘no’ for the interaction between conducting and participating in fire drills (top) compared to the other interactions. Interestingly, the interaction for conducting fire drills: yes and participating in fire drills: no (top) had the highest median for respondents as compared to other interactions (Figure 1).

For the model of maintenance and recordkeeping, the number of responses to the questions about keeping records of all maintenance (Wald χ^2^ = 11.493; *p* = 0.00) and the frequency of fire safety equipment inspection and maintenance (Wald χ^2^ = 25.424; *p* = 0.001) showed statistically significant results. However, the question of the knowledge of the existence of electrical compliance certificates (Wald χ^2^ = 2.929; *p* = 0.086) and the mode of recording of participation in fire evacuation drills (Wald χ^2^ = 8.733; *p* = 0.120) did not predict the number of responses for these questions.

**FIGURE 2:**
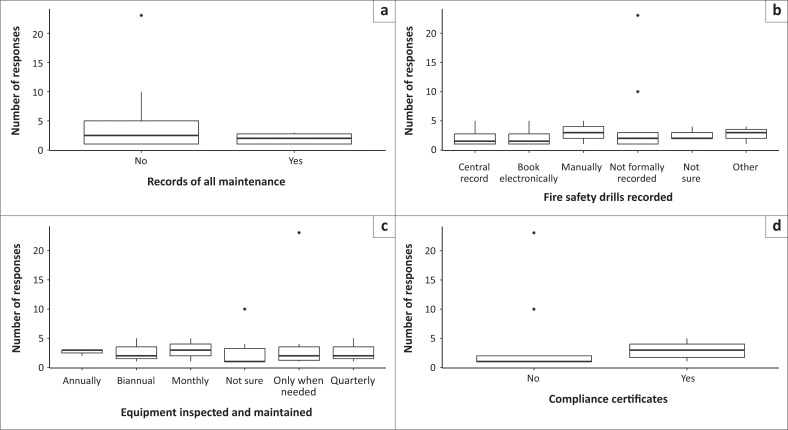
Number of responses for model maintenance and recordkeeping: Records of all maintenance. (a) fire safety drills recorded; (b) equipment inspected and maintained; (c) compliance certificate; (d) boxes show the first and third quartiles and medians (solid black line across the box). Whiskers show total range, and dots outside of boxes indicate outliers.

The most frequent responses were ‘no’ across the predictor variables as follows: (1) The questions of whether the schools are keeping records of all maintenance (top left); (2) The question about the mode in which fire drills are recorded manually (top right); (3) The question on the frequency of fire safety equipment inspection and maintenance which most respondents reported monthly (bottom left); and (4) Most respondents reported compliance certificates (bottom right; Figure 2).

In the third model of staff knowledge of protocol, the questions of staff’s knowledge of their responsibility (Wald χ^2^ = 33.088; *p* = 0.001) and whether staff will be able to assist with evacuation (Wald χ^2^ = 8.385; *p* = 0.001) were significant predictors of the number of responses. However, staff’s knowledge of step-by-step fire evacuation procedure (Wald χ^2^ = 4.439; *p* = 0.139), specific to their school should a fire break out and the knowledge of operating a fire extinguisher (Wald χ^2^ = 49.160; *p* = 0.212) did not significantly predict the number of responses.

**FIGURE 3:**
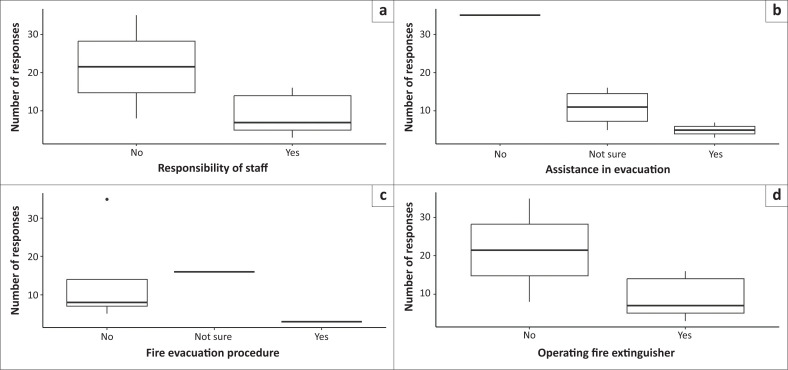
Number of responses for model staff’s knowledge of their responsibility: The responsibility of staff. (a) assistance with evacuation; (b) fire evacuation procedure; (c) operating fire extinguisher; (d) boxes show the first and third quartiles and medians (solid black line across the box). Whiskers show total range, and dots outside of boxes indicate outliers.

Most respondents reported ‘no’ when asked if staff knew their responsibility should a fire break out in their school (top left). Also, most of the respondents were ‘not sure’ whether they would be able to assist with evacuation should a fire break out in their school (top right). In addition, the majority of the respondents reported ‘no’ when asked if they would be able to assist with evacuation procedures following their training should a fire break out in their schools (bottom left). Furthermore, the majority of the respondents reported ‘no’ when they were asked if they knew how to operate a fire extinguisher (bottom right; Figure 3).

When respondents were asked if they were aware of the existence of fire safety plans in their schools to prepare for fires, most respondents (*n* = 44) were not sure, 35 reported no, and only 9 reported yes.

The study then applied a GLM for fire compliance, which revealed a significant difference in response to the question of the availability of protocols for handling flammable chemicals (Wald χ^2^ = 18.914; *p* = 0.050) only. There were no significant differences shown for the awareness of the existence or recruitment of fire safety officers or safety committees (Wald χ^2^ = 18.914; *p* = 0.260); the use of any form of flammable chemicals (Wald χ^2^ = 17.647; *p* = 0.057); storage of flammable chemicals in purpose-made storage (Wald χ^2^ = 16.792; *p* = 0.999); registrations of flammable chemicals with the local fire services (Wald χ^2^ = 16.049; *p* = 0.917) and the use of gas used in your school premises (Wald χ^2^ = 16.744; *p* = 0.989).

**FIGURE 4:**
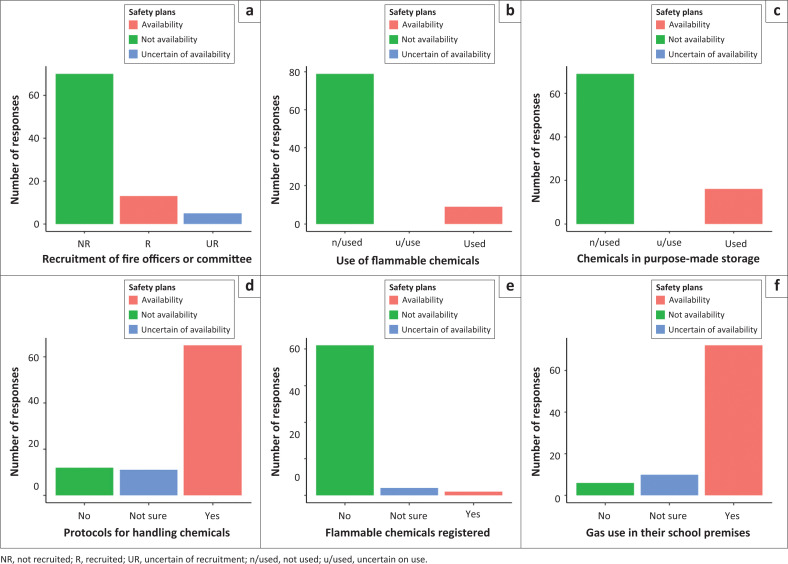
The number of reports about the fire safety plans (available; not available; uncertain of availability) reported by respondents for recruitment of fire officers or committee. (a) use of flammable chemicals; (b) chemicals stored in purpose-made storage; (c) protocols for handling chemicals; (d) flammable chemicals registered; (e) use of gas in their school premises; (f) from the sampled special needs schools in the North West Province of South Africa.

Overall, more respondents reported the unavailability of safety plans compared to those who reported their availability or were uncertain about it across all predictor variables (Figure 4). The majority of the respondents who reported the unavailability of safety plans in their schools also reported that their schools did recruit fire safety officers or committees and that their schools did not register their flammable chemicals with the local fire services (Figure 4).

### School fire safety management: Disaster Preparedness Strategy framework

The regression results showed a significant relationship when the question of whether the schools engage in community-based disaster preparedness (dependent variable) was regressed with other eight Likert scale questions (χ^2^ = 206.7; *df* = 8; *p* = 0.01; *R*^2^ = 90.5%). Furthermore, a significant difference was found in the question of whether the schools have Early Warning System (EWS) only (Table 1).

## Discussion

The study assesses the fire preparedness of nine SNS in the North West Province of South Africa and starts by discussing fire hazard preparedness in the context of the findings.

This section discusses only the results that were statistically significant for the four GLM and regression models applied for this study and the implications of these results based on the existing literature. The statistically significant differences found for the question of whether the schools conducted fire drills, whether the staff participated in their school’s fire drills, and the staff’s confidence in their ability to assist students with special needs during a fire evacuation after receiving the school’s fire preparedness training were important because participation in these drills and the confidence of these trained staff are important determinants of fire preparedness (Ooi, Tanimoto & Sano 2019; Ryan et al. 2020; Vásquez et al. 2018).

These results are also consistent with other studies. In Japan, a study by Ooi et al. (2019) reported the current state of disaster prevention education in schools involves people who are trained in fire drills and are aware of the necessity of education and training for disaster preparedness. Another study in Iquique City in the Tarapacá Region, in Northern Chile, reported children’s views on evacuation drills and school preparedness were mostly positive (Vásquez et al. 2018). Indeed, effective preparedness for fire drills has been reported to require confidence from those who are being trained, as these trainings include a wide range of techniques that work together and might need some level of confidence (Ryan et al. 2020).

The highest median for yes/no responses for the interaction between conducting and participating in fire drills found by the study was worrisome. While these results show that the sampled schools are conducting some fire drills, there seems to be a lack of interest to participate, as the highest number of respondents reported ‘no’ to participation in these drills.

The significant differences found in the number of responses to the questions about keeping records of all maintenance were consistent with the requirement of the *South African Disaster Management Act* (DMA). The *DMA Act 15 of 2016* is mandating recordkeeping for effective disaster management and for better preparedness to save lives, especially the lives of the most vulnerable, which includes people with disabilities and special needs.

In addition, most respondents reported ‘no’ when asked if their schools keep records of all maintenance and that the schools keep compliance certificates (Figure 2). Recordkeeping and compliance certificates are some of the determinants of fire hazard preparedness, which also entails covering all bases to avoid litigations, especially in SNS setups.

The questions of staff’s knowledge of their responsibility and whether staff will be able to assist with evacuation significantly predicted the number of responses. However, most respondents reported ‘no’ when asked if staff knew their responsibility should a fire break out in their school, and the majority responded ‘not sure’ regarding whether staff will be able to assist with evacuation should a fire break out in their schools.

The finding that the majority of the respondents report that staff do not know their responsibility should a fire break out in their schools has significant implications in the context of disaster preparedness. These implications include increased risk, as a lack of knowledge about fire emergency procedures significantly increases the risk to both students and staff (Kihila 2017). In a SNS, students may have varying levels of mobility, communication skills and understanding of emergencies, necessitating well-coordinated and informed staff responses (Tarricone, Mestan & Teo 2021).

The results indicating that a majority of respondents (*n* = 44) were unsure about the existence of fire safety plans in their schools have several significant implications in the context of disaster preparedness and management policies in South Africa, especially concerning SNS. The results reflect possible shortcomings in the implementation of disaster preparedness policies within the basic education sector. While policies might exist on paper, their practical application and enforcement in the sampled SNS appear to be lacking as respondents cannot be uncertain if the policy is implemented. Indeed, disaster management plans are mandated by the *South African Disaster Management Act* for each organ of the state, including the Education Sector.

The statistically significant result indicating uncertainty about staff’s ability to assist with evacuation in the event of a fire highlights critical areas such as training and communication needing attention in disaster preparedness across the sampled schools. Indeed, communication and training are a big part of disaster preparedness across the world (Dufty 2020). The GLM findings showing a significant difference in the response to the question of the availability of protocols for handling flammable chemicals suggest a need for stricter regulations and oversight regarding fire safety and the handling of flammable chemicals in sampled SNS.

The ordinal regression indicating a significant difference for early warning systems suggests that only the sampled schools with Early Warning Systems (EWS) in place will have different levels of preparedness compared to those without EWS. For example, schools with EWS might show higher levels of preparedness, indicating that EWS contributes positively to preparedness measures.

## Conclusion

In conclusion, the study sheds light on the current state of disaster preparedness, specifically focusing on fire-related incidents, within the sampled schools. This finding of the majority of staff not knowing their responsibility should a fire break out in their school highlights the need for more detailed research into the specific challenges and barriers to effective disaster preparedness in SNS. This study recommends a comparative future study, comparing the preparedness levels and strategies of different schools, both within South African provinces (as the North West Province is predominantly rural; StatsSA 2022) and internationally, which could provide valuable insights and best practices that can be adapted and implemented at the sampled schools.

Addressing training, communication, policy review and stakeholder engagement can help improve preparedness and ensure the safety and well-being of students and staff in SNS and also ensure the staff’s ability to assist with evacuation with confidence during fire breakouts.

The majority of uncertain respondents about the existence of fire disaster management plans for the sampled schools could indicate resource allocation issues. There may be inadequate resources allocated to implementing and maintaining effective fire safety measures in the sampled SNS, and while a plan is in place, it has not been updated or implemented effectively.

The GLM showing a significant difference in the response to the question of the availability of protocols for handling flammable chemicals reveals an enhanced oversight by the school management when it comes to disaster preparedness. Policymakers may need to develop or update existing regulations to ensure these protocols are standardised and enforced in these government schools. Because of the insignificant results found for other parameters from the framework, there needs to be an improvement by the sampled schools in terms of hazard, risk, and vulnerability assessment, response mechanisms and strategies, fire preparedness plans, fire coordinators’ effective information system and how the sampled schools mobilise their resources and engage in public education, training and rehearsals, as this will assist the schools with their Disaster Preparedness Strategy framework.

Overall, the study highlights areas for improvement in disaster preparedness within the sampled schools, emphasising the importance of adequate safety measures to mitigate the risks associated with fire incidents. The findings could serve as a valuable resource for policymakers, educators and school administrators to enhance the safety and resilience of educational institutions in the face of potential disasters.
